# Human Mast Cell Tryptase Is a Potential Treatment for Snakebite Envenoming Across Multiple Snake Species

**DOI:** 10.3389/fimmu.2018.01532

**Published:** 2018-07-09

**Authors:** Elizabeth Anderson, Kathrin Stavenhagen, Daniel Kolarich, Christian P. Sommerhoff, Marcus Maurer, Martin Metz

**Affiliations:** ^1^Department of Dermatology and Allergy, Charité – Universitätsmedizin Berlin, Berlin, Germany; ^2^Department of Biomolecular Systems, Max Planck Institute of Colloids and Interfaces, Potsdam, Germany; ^3^Institute of Laboratory Medicine, University Hospital, Ludwig-Maximilians-University, Munich, Germany

**Keywords:** mast cell, venom, proteases, snakes, antivenom

## Abstract

Snake envenoming is a serious and neglected public health crisis that is responsible for as many as 125,000 deaths per year, which is one of the reasons the World Health Organization has recently reinstated snakebite envenoming to its list of category A neglected tropical diseases. Here, we investigated the ability of human mast cell proteases to detoxify six venoms from a spectrum of phylogenetically distinct snakes. To this end, we developed a zebrafish model to assess effects on the toxicity of the venoms and characterized the degradation of venom proteins by mass spectrometry. All snake venoms tested were detoxified by degradation of various venom proteins by the mast cell protease tryptase β, and not by other proteases. Our data show that recombinant human tryptase β degrades and detoxifies a phylogenetically wide range of venoms, indicating that recombinant human tryptase could possibly be developed as a universal antidote to venomous snakebites.

## Introduction

The development of affordable and effective non-species-specific antivenom could dramatically reduce the global burden of snakebite (1). Yearly, up to 2,500,000 cases result in as many as 125,000 deaths and 400,000 amputations worldwide, with by far the highest rates occurring in poor and rural areas of South and Southeast Asia, sub-Saharan Africa, and Oceania (1–4). In addition to the morbidity and mortality, the economic burden of snakebites is considerable (5), especially as most victims of snakebite live in the world’s poorest communities, with agricultural workers, children, and rural dwellers most at risk (6).

Current treatments for snakebite are problematic for several reasons. Apart from political and economic factors that have led to a dramatic loss of antivenom manufacturers and insufficient product regulation in Africa, Asia, and Oceania (7), there are a number of technical problems inherent in this treatment. For example, antisera are species-specific, proper usage requires both accurate species identification and availability of the corresponding antivenom, and they must be administered intravenously at often difficult to reach clinics. Even then, antibody sera come with an inherent risk of adverse immune side effects, including anaphylactic shock (8–10). For example, more than 50% of patients receiving a horse-derived antivenom developed early adverse reaction to the antivenom with 30% experiencing severe anaphylaxis (11). Thus, a non-species-specific treatment that could be immediately and locally administered without the risk of adverse immune reactions would be a significant improvement.

We and others have recently shown that in mice, mast cells (MCs) can limit the toxicity of animal venoms by releasing proteases that degrade and thereby detoxify venom components. The proteases carboxypeptidase A (CPA) and chymase mouse MC protease-4 were found to be responsible for the protective effects of MC against various animal venoms (12–14). To date, the role of human MC-derived proteases in responses to snake venoms has not been reported. If protective, these proteases could be developed as a novel local treatment for envenoming by a broad range of snake species.

## Materials and Methods

### Mast Cells

The MC line LAD2 was kindly provided from Dr. Arnold S. Kirshenbaum (National Institute of Allergy and Infectious Diseases, Bethesda, MD, USA) and cultured as described (15).

Human skin mast cells (hsMCs) were isolated according to a published protocol (16) with modifications as reviewed and approved by the Charité—Universitätsmedizin Berlin Institutional Review Board. Human abdominal skin from healthy adults undergoing abdominoplasty was cut into strips and treated with Dispase Type I (Roche Diagnostics) at 2.4 U/ml and 4°C overnight. The dermis was cut into small pieces and digested with collagenase (Worthington) at 10 mg/g of tissue, hyaluronidase (Sigma-Aldrich) at 5–7.5 mg/g, and 10 µg/ml DNAse (Roche Diagnostics) suspended in 10 ml of dispersing medium per g (PBS, 1% penicillin/streptomycin, 5% FCS, 1.5 µg/ml Amphotericin B, 5 mM MgSO_4_) for 1 h at 37°C. The isolated cells were separated from the remaining tissue by three steps of filtration (pore sizes 100, 40, and 20 µm) and the digestion was repeated one to two times. MCs were further purified from the suspension by positive selection using anti-CD117 magnetic microbeads (Miltenyi) and a magnetic cell sorter. MC purity in these separations typically exceeded 95%, as assessed by Kimura staining. Informed consent of the patients has been obtained.

### β-Hexosaminidase Release Assay

We stimulated a total of 1.5 × 10^5^ LAD2 MC or 0.6–1.5 × 10^5^ purified hsMCs in Tyrode’s buffer [10 mM HEPES buffer (pH 7.4), 130 mM NaCl, 5 mM KCl, 1 mM MgCl_2_, 1.4 mM CaCl_2_, 5.6 mM glucose, and 0.1% BSA] with the indicated concentrations of snake venom or 1 µM calcium ionomycin (Sigma-Aldrich) for 30 min at 37°C and performed β-hexosaminidase release assay as previously described by us and others (14, 17–19). In brief, after stimulation with venoms, cells were separated by centrifugation and β-hexosaminidase levels were measured in both supernatants and in cell pellets. MC degranulation was then assessed as percentage release of total β-hexosaminidase.

### Venoms and Proteases

Venoms from *Agkistrodon contortrix contortrix* (southern copperhead), *Crotalus atrox* (western diamondback rattlesnake), and *Echis carinatus* (saw-scaled viper) were purchased from Sigma-Aldrich; venoms from *Bothrops atrox* (common lancehead), *Daboia russelii* (Russell’s viper), and *Naja pallida* (red spitting cobra) from Latoxan, France. Purified human lung tryptase, purified human pancreas CPA, and purified human skin chymase were purchased from Elastin Products Company, MO, USA.

Venom concentrations used for MC stimulation and zebrafish survival assays were based on published LD50s, adjusted for the respective experimental setting. Final concentrations for MC activation were 125 µg/ml common lancehead; 25 µg/ml saw-scaled viper; 30 µg/ml southern copperhead; 7.5 µg/ml red spitting cobra; 20 µg/ml Russell’s viper; and 200 µg/ml western diamondback rattlesnake. Final concentrations for zebrafish assays were 1,000 µg/ml common lancehead; 200 µg/ml saw-scaled viper; 400 µg/ml southern copperhead; 6.25 µg/ml red spitting cobra; 300 µg/ml Russell’s viper; and 1,000 µg/ml western diamondback rattlesnake.

### Recombinant Tryptase β

Recombinant human tryptase β was expressed in *Pichia pastoris* and isolated by a modification of published methods (20, 21). The purity of the recombinant protease was ≥95% as determined by SDS-PAGE and N-terminal amino acid sequencing. The specific activity, quantified by both burst titration with 4-methylumbelliferyl *p*-guanidinobenzoate and titration with synthetic inhibitors (22), was ≥90% of the theoretical value. Inactivated tryptase was prepared by reaction with 4-(2-Aminoethyl)-benzenesulfonyl fluoride hydrochloride (Roche Diagnostics) and subsequent removal of residual reagents. Recombinant human tryptase was stabilized routinely with unfractionated heparin from bovine intestinal mucosa (Sigma-Aldrich).

### Zebrafish Survival Assays

Wild-type Zoltan zebrafish were provided by Salim Seyfried, Max-Delbrück-Center for Molecular Medicine, Berlin. Zebrafish maintenance and embryo collection were carried out according to the standard practice. At 48 h post fertilization (hpf), embryos were transported to our lab for same-day use shortly after hatching. Fish were kept at 27°C. Assays were performed at 20–25°C.

For the survival experiments, zebrafish embryos were individually suspended in 50 µl of egg water in a 96-well microtiter plate and 50 µl venom/treatment solutions were added for final volume of 100 µl. For assays of hsMC lysate treatment, freshly purified hsMCs were resuspended in PBS, lysed by ultrasonication and stored at −80°C. Lysate from four different donors was pooled and diluted in PBS, and incubated at various concentrations with venom for 60 min at 37°C, which was then transferred to microwells containing single zebrafish for toxicity testing. Likewise, purified human tryptase, chymase, CPA, or recombinant tryptase β were first combined in PBS and incubated at 37°C for 60 min at indicated concentrations before exposure to embryos.

To test hsMC lysate or purified human tryptase treatments given at short time intervals after initial venom exposure, venom alone (also incubated at 37°C for 60 min prior to use) was added to microwells as above. Subsequently, 10 µl of a 10× concentrated solution of lysate or tryptase treatment was added to the wells at the indicated time points.

Controls were prepared with identical concentrations of venom, enzyme, lysate, or PBS alone. Active recombinant tryptase β contained a ratio of 12:1 heparin to tryptase for tetramer stabilization. Zebrafish were monitored at regular intervals at 40× magnification using a standard inverted microscope. Death was defined as cessation of the heartbeat, which is clearly visible through the transparent skin at this stage of development. Zebrafish that survived at least 6 h were generally observed to remain alive at least 24 h.

### Mass Spectrometry (MS) Analysis

Samples were prepared at a ratio of 10^3^:1 venom to tryptase by mass (southern copperhead and common lancehead) or 10:1 venom to tryptase by mass (western diamondback rattlesnake, Russell’s viper, saw-scaled viper, red spitting cobra) in PBS, incubated at 37°C for 30 min, and stored at −20°C. Controls were prepared with venom and PBS alone.

### MALDI-TOF-MS Analysis

MALDI-TOF-MS analyses were performed on an AutoflexTM Speed mass spectrometer using an AnchorChip (800/384) MALDI target plate (Bruker Daltonics, Bremen, Germany). Mass spectra were acquired in positive ion reflectron mode within the mass range of *m/z* 3,500 to *m/z* 18,000, collecting 10,000 shots (smartbeam™-II laser) with constant laser intensity, averaged over the entire spot. External calibration of the TOF analyzer was performed prior the first analysis of a batch using protein calibration standard I (Bruker Daltonics, Bremen, Germany).

Prior to MALDI-TOF-MS analyses, 17 µl of the venom sample (in PBS) were purified by reversed phase chromatography using C18 Zip TipsTM (Millipore). The tips were flushed with 70% acetonitril (ACN) containing 0.1% trifluoroacetic acid (TFA) and equilibrated with 5% ACN/0.1% TFA prior sample purification. The sample was applied to the tip by repeated pipetting (20 times) according to the manufacturer’s instructions. Bound peptides/proteins were desalted by washing the tip five times with 20 µl of 5% ACN/0.1% TFA before the bound components were eluted with 11 µl of 70% ACN/0.1% TFA.

Equal volumes (2 µl each) of the individually purified samples were mixed with 2 µl of horse heart myoglobin (1 µg/µl) as an internal reference. One microliter of the mix followed by another microliter of 2,5-dihydroxybenzoic acid matrix (DHB, 10 mg/ml in 30% ACN/0.1% TFA, Sigma-Aldrich) were spotted onto the MALDI target plate and dried under ambient conditions at RT.

The acquired mass spectra were manually analyzed using DataAnalysis 4.0 (Bruker Daltonics). Five intense signals distributed over the acquired mass range were selected and their signal intensities normalized to the intensity of the doubly charged signal of myoglobin (*m/z* 8,476) to semi-quantitatively evaluate the change in signal intensities by tryptase treatment. Further detail is provided in the Protein Report in Supplementary Material.

### Reversed Phase LC-ESI-MS/MS

Samples were analyzed by RP-LC-ESI-MS/MS on an amaZon ETD ion trap (Bruker Daltonics, Bremen, Germany) coupled to an Ultimate 3000 UHPLC system (Dionex, Part of Thermo Fisher) as described previously, with minor modifications (23). Peptide MS/MS spectra were searched against the SwissProt database using ProteinScape 3.1 (Bruker Daltonics, Bremen, Germany) and MASCOT 2.3 (MatrixScience, London, UK) using the following search parameters in the UniProt database (Release 2011_08 and reanalyzed with 2013_08): deamidation (Asn/Gln) and oxidation (Met) were set as variable modifications and chordata was chosen as taxonomy. Up to nine missed cleavages were allowed selecting trypsin as acting enzyme. The high number of missed cleavages was used since tryptase cleaves after the same amino acids as trypsin but with higher selectivity. Peptide tolerance (both MS and MS/MS) was set at ±0.2 Da.

## Results

### Snake Venom Induces Human Mast Cell Degranulation

**Figure 1:**
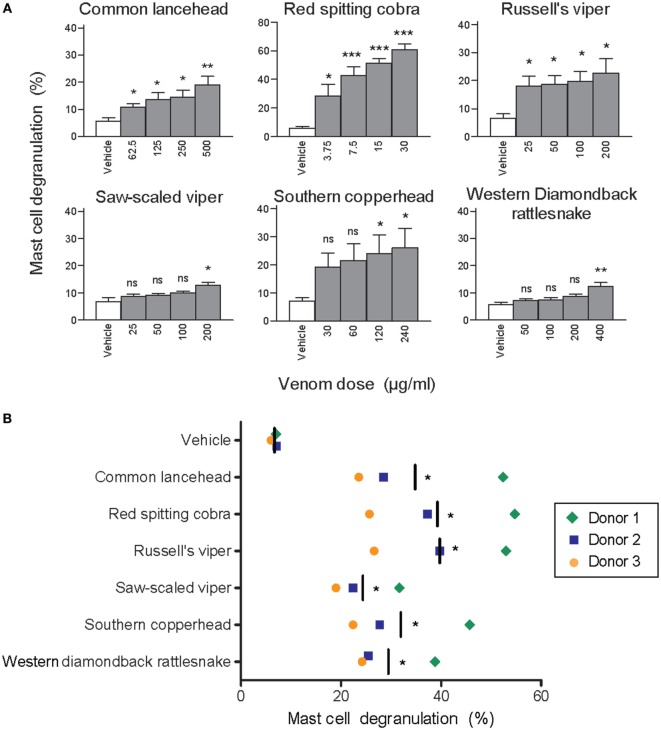
Whole venom from six different snakes induces degranulation of human mast cells. LAD2 MCs **(A)** or primary human skin mast cell (hsMCs) isolated from three healthy donors **(B)** were stimulated with venom from the six listed snake species. **(B)** Venom doses were as follows: common lancehead, saw-scaled viper, southern copperhead, red spitting cobra, Russell’s viper, and western diamondback rattlesnake. Negative controls (vehicle) were PBS only. Positive controls (not shown) were Ca ionomycin (1 µM) and caused an average of 55% degranulation in LAD2 **(A)**, and 62, 36, and 8% degranulation in hsMCs from each donor **(B)**. Data are pooled from four independent experiments and measurements were performed in triplicate. Student’s *t*-test compares venom to vehicle stimulation. *p < 0.05; **p < 0.01; ***p < 0.001.

To test whether clinically relevant snake venoms can activate human MCs we first stimulated LAD2 cells with venom from six taxonomically and geographically diverse venomous snakes, which are all listed by the World Health Organization as snakes with a high medical importance in their respective region (1, 24–26). All venoms induced dose-dependent MC degranulation as assessed by β-hexosaminidase release (Figure 1A). Since LAD2 cells differ considerably from primary hsMCs [for example, they contain comparatively low levels of proteases (27)] we also tested freshly isolated and purified hsMCs. Skin MCs from three individual healthy donors degranulated in the presence of all tested venoms (Figure 1B).

### Human Mast Cells Protect Zebrafish Embryos From Lethal Doses of Snake Venom

**Figure 2:**
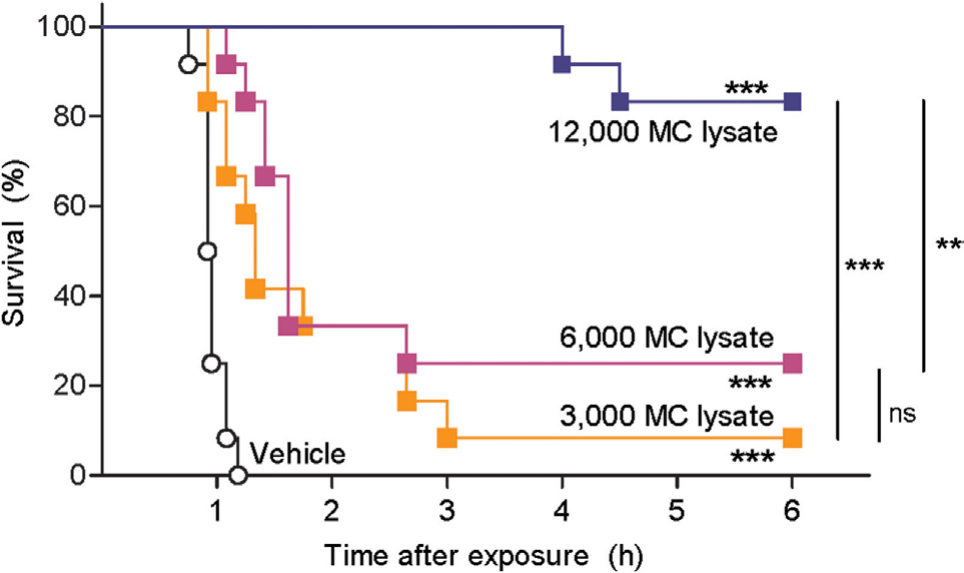
Lysate from purified human skin mast cells (hsMCs) protects zebrafish from toxicity of cobra venom. Zebrafish embryos aged 48 h post fertilization suspended in 100 µl of fish water received 625 ng red spitting cobra whole venom pre-treated with lysate of purified hsMCs (MC lysate) or PBS (vehicle). Lysate doses were 12-, 6-, or 3- thousand cells per fish. Survival was monitored at regular intervals for 6 h, with complete cessation of heartbeat as the predefined endpoint. Vehicle, lysate alone, or enzyme alone had no effect (data not shown). Data are pooled from three independent experiments, *n* = 12 fish per group. Kaplan–Meyer survival analysis, Log-rank (Mantel-Cox) test compares treatments to vehicle and to each other. **p < 0.01; ***p < 0.001.

Next, we tested whether hsMC can neutralize the toxic effects of snake venom. Traditionally, venom toxicity is quantified as the amount required to kill 50% of a test population of mice, i.e. the lethal dose, 50% (LD50) (28). To avoid using an unethically high number of mice, we developed an alternative model for the assessment of the biological activity of venoms using zebrafish embryos at 48 hpf. With this model, we first measured rates of survival of zebrafish embryos exposed to red spitting cobra venom that had or had not been treated with lysates of purified hsMCs. Lethality of the cobra venom was markedly reduced when the venom was treated with MC lysates from as little as 3,000 MCs per fish (Figure 2). Lysates from larger numbers of MCs resulted in increased survival rates of the zebrafish embryos, with almost complete protection after incubation with lysate from 12,000 MC (Figure 2).

### Purified Human Tryptase Detoxifies Snake Venom

**Figure 3:**
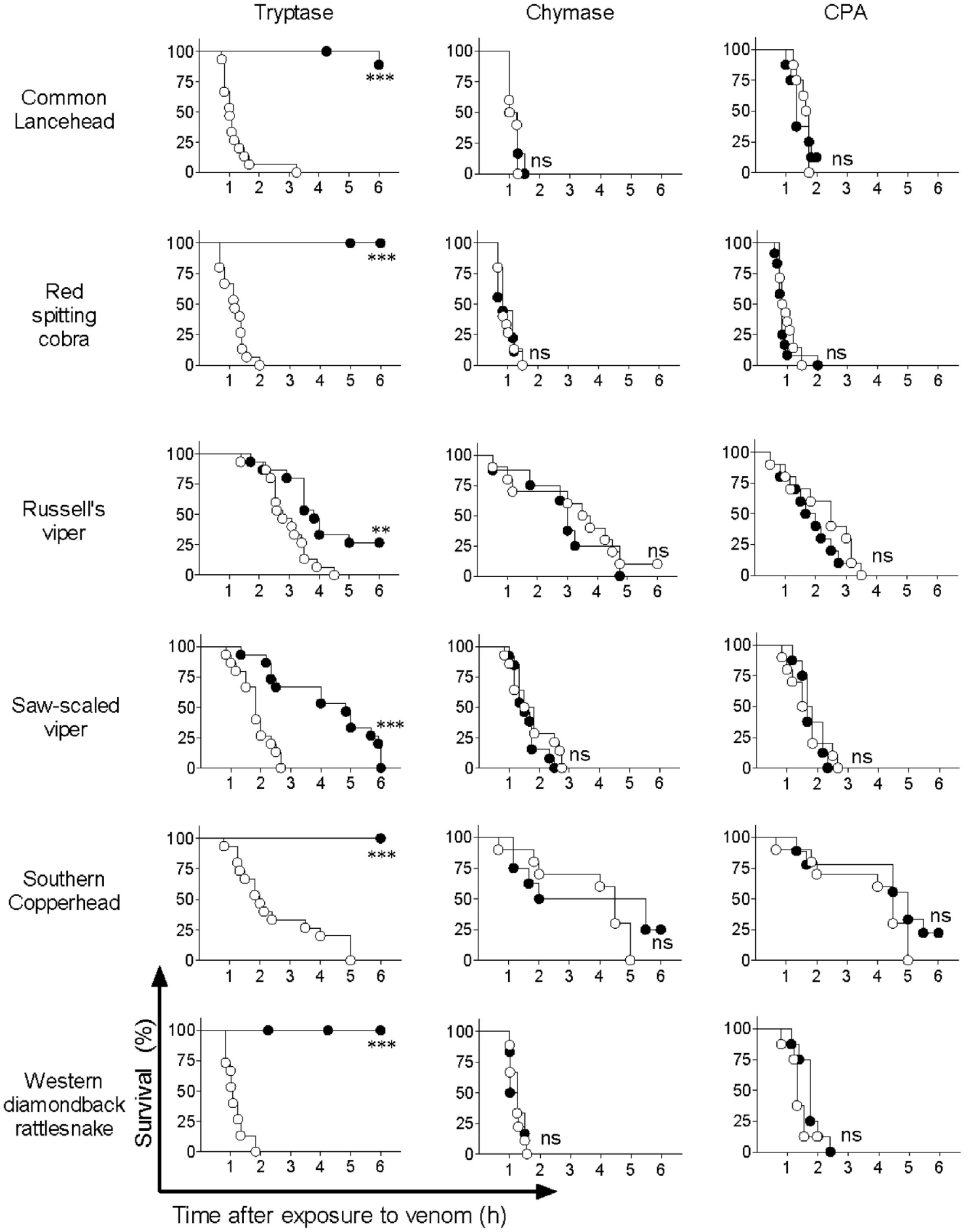
Purified human tryptase, but not chymase or carboxypeptidase A (CPA), detoxifies snake venoms. Venoms were incubated with 10 µg/ml human tryptase, chymase, or CPA. Treated and untreated venom were then administered to 48 h post fertilization zebrafish in 100 µl of water. Empty circles represent zebrafish receiving venom alone, and solid shapes represent fish receiving enzyme-treated venom. Vehicle or enzyme alone had no effect (data not shown). Data are pooled from three (tryptase) or two (chymase and CPA) independent experiments for *n* = 15 or 10 fish per group. Kaplan–Meyer survival analysis, Log-rank (Mantel-Cox) test compares treatments to vehicle. **p < 0.01; ***p < 0.001.

Based on previous reports by us and others showing that mouse MC proteases are able to detoxify snake venom or snake venom components (13, 29), we hypothesized that the observed detoxification of red spitting cobra venom by MC lysates is mediated by MC-derived proteases. To verify this, and to assess whether other snake venoms can also be detoxified by MC proteases, we tested three prominent human MC proteases—tryptase, chymase, and CPA—for protective effects in the zebrafish model. Purified human skin chymase and purified human pancreatic CPA had no protective effect against any of the venoms (Figure 3), while both enzymes were confirmed to be active by degradation of specific chromogenic substrates (data not shown). By contrast, purified human lung tryptase completely protected zebrafish embryos from the lethal effects of common lancehead, red spitting cobra, southern copperhead, and western diamondback rattlesnake venom and significantly improved survival of embryos exposed to Russell’s viper and saw-scaled viper venoms (Figure 3).

### Mast Cell Tryptase Degrades Snake Venom Toxins

**Figure 4:**
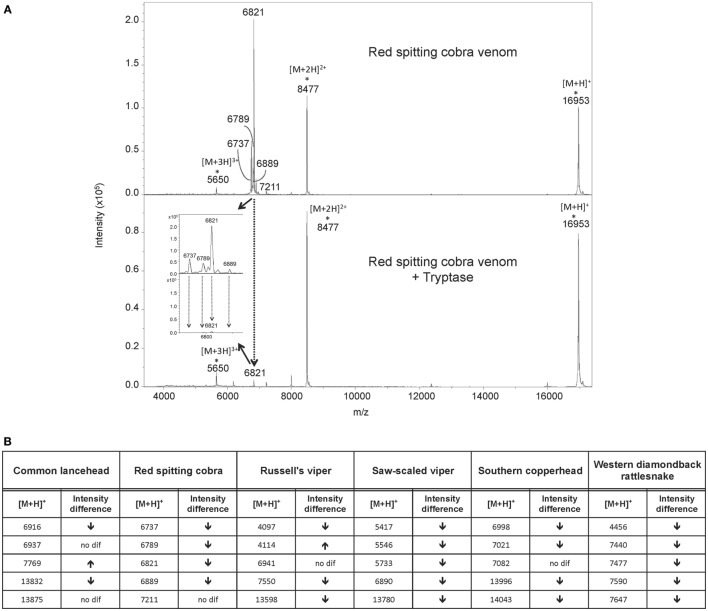
MALDI-TOF-MS analysis of venoms treated with purified human tryptase shows degradation of venom proteins. Samples were prepared at concentrations of 10 µg/µl venom and a ratio of 1000:1 venom to tryptase by mass (southern copperhead and common lancehead) or 0.1 µg/µl and a ratio of 10:1 venom to tryptase by mass (western diamondback rattlesnake, Russell’s viper, saw-scaled viper, and red spitting cobra). They were then incubated at 37°C for 30 min, and stored at −20°C. Control was prepared with venom and PBS alone. 17 µl of the venom sample (in PBS) was purified by reversed phase chromatography using C18 Zip TipsTM (Millipore). Mass spectra were acquired in positive ion reflectron mode within the *m/z* ratio range of *m/z* 3,500 to *m/z* 18,000. Panel **(A)** shows a representative MALDI-TOF-MS spectrum of red spitting cobra venom before and after incubation with purified human tryptase. Five signals (dashed arrows) over the acquired mass range were selected and their signal intensities normalized to the intensity of the doubly charged myoglobin signal (*m/z* 8,477). The relative signal intensity reduction of several peaks indicates protein degradation by tryptase. Spectra for additional venoms are provided in Figure 5. Comparisons of relative signal intensities for all six venoms are summarized in **(B)**. Arrows indicate a reduced (↓) or increased (↑) relative signal intensity difference (Intens. dif.) of prominent peaks, or no difference (no dif.), with lower peaks indicating degradation of the polypeptide of the corresponding *m/z* ratio.

**Figure 5:**
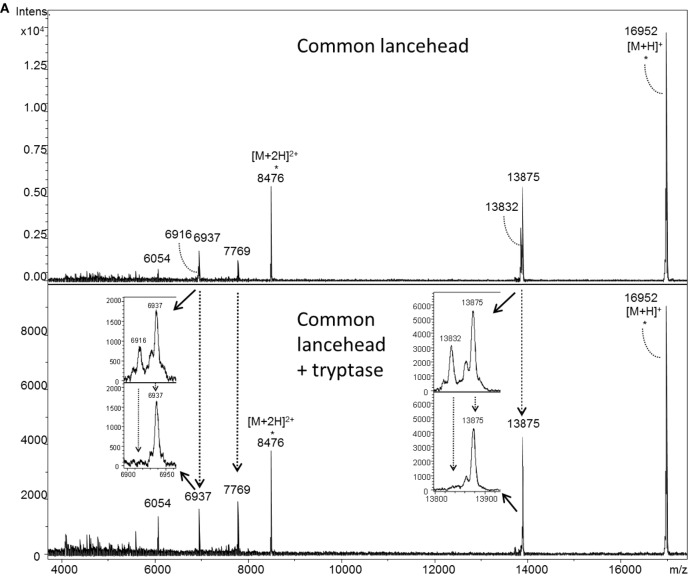

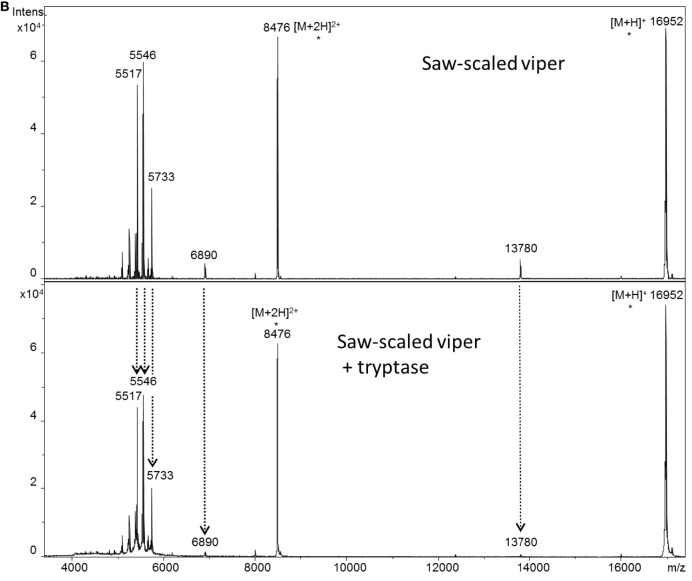

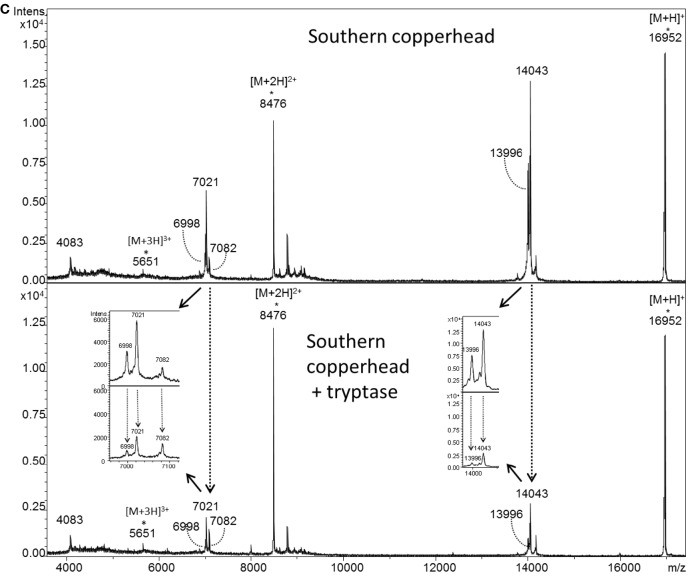

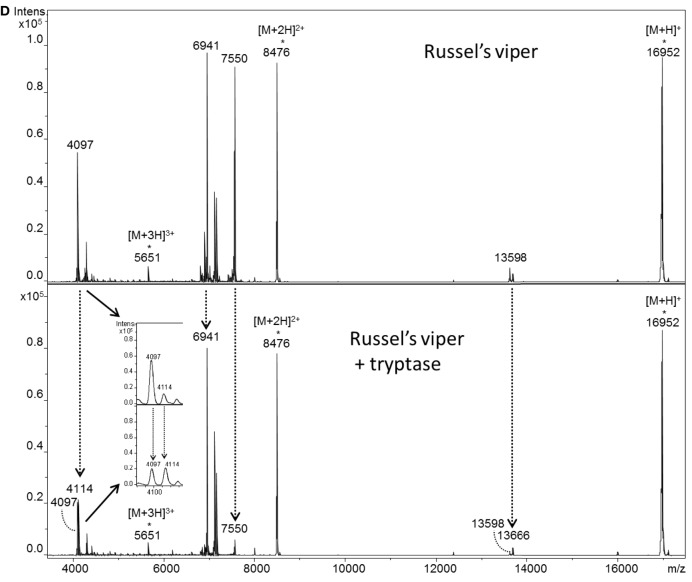

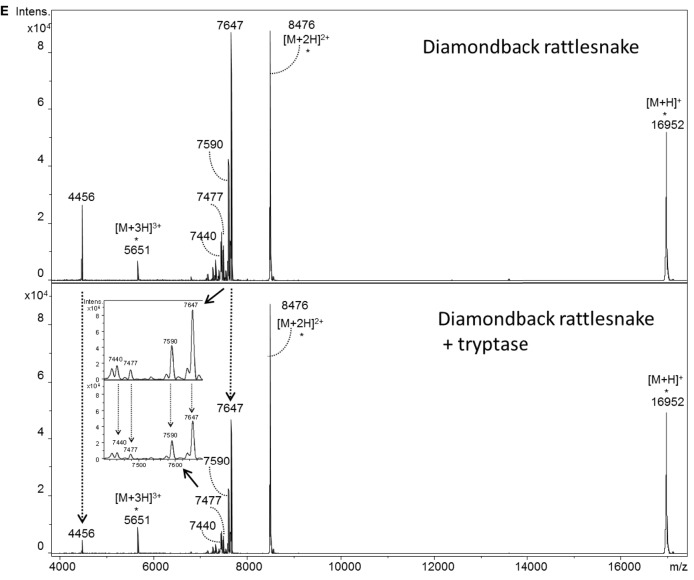
MALDI-TOF-MS analyses of whole venoms treated with purified human tryptase shows degradation of venom proteins. Venoms from five snake species [(**A**) common lancehead, **(B)** saw-scaled viper, (**C**) southern copperhead, (**D**) russel’s viper, **(E)**, western diamondback rattlesnake] were treated with tryptase or vehicle control. Spectra were acquired within the mass range of *m/z* 3,500 to *m/z* 18,000. Samples contain equal amounts (0.5 µg) myoglobin as an internal reference (*m/z* 16,952 [M + H]^+^, *m/z* 8,476 [M + 2H]^2+^, *m/z* 5,651 [M + 3H]^3+^). Venom-derived signals (dashed arrows) distributed over the whole mass range were selected and normalized to the intensity of the doubly charged myoglobin signal (Figure 4B). The reduction in relative signal intensities of several peaks in tryptase-treated versus untreated venom indicates tryptase-mediated protein degradation.

To investigate how tryptase provides protection from the toxic effects of snake venoms, we used MS to compare tryptase-treated and untreated venom from cobra (Figure 4A) and five other snakes (Figure 5). MALDI-TOF-MS analyses showed reduction in the intensities of 23 out of 30 signals for venom proteins after treatment with purified human tryptase (Figure 4B). The loss of numerous signature venom components in all six snake species tested suggests degradation by tryptase.

To verify that proteins with decreased signal intensity had in fact been degraded, we used reversed phase LC-ESI-MS/MS analysis to test for small peptide fragments in tryptase-treated and untreated venoms. Peptides derived from l-amino-acid oxidase were identified in five venoms after tryptase treatment, but not in the control samples, indicating degradation by tryptase (Table 1). The high peptide sequence homology (30) allowed for successful identification of this protein even in species where only closely related protein sequences are available (southern copperhead and common lancehead).

**Table 1:** Venom protein identification using LC-ESI-MS/MS reveals peptide fragments of known venom toxins in samples treated with purified human tryptase.

	Type of fragmentation	UniProt entry name	Protein name	Protein mass (kDa)	Observed peptide (*m/z*)	Charge state	Mass difference (ppm)	Peptide sequence	Protein score
Southern copperhead + tryptase	CID	OXLA_BOTJR	l-amino-acid oxidase (fragment) OS = *Bothrops jararacussu* PE = 1 SV = 1	56.30	441.34	3	155.90	RIKFEPPLPPK	70.21
					432.00	3	148.58	IKFEPPLPPKK	

Common lancehead + tryptase	CID	OXLA_BOTJR	l-amino-acid oxidase (fragment) OS = *Bothrops jararacussu* PE = 1 SV = 1	56.30	441.35	3	178.56	RIKFEPPLPPK	146.69
					463.60	3	90.68	KFWEDDGIHGGK	
					420.93	3	167.17	FWEDDGIHGGK	
					438.78	2	126.03	STTDLPSR	

Western diamondback rattlesnake + tryptase	CID	OXLA_CROAD	l-amino-acid oxidase OS = *Crotalus adamanteus* PE = 1 SV = 1	58.60	438.68	2	−11.11	VIEIQQNDR	65.46
					557.79	2	−101.90	STTDLPSR	
	CID	VM1AC_CROAT	Snake venom metalloproteinase atrolysin-C OS = *Crotalus atrox* PE = 1 SV = 1	46.70	548.24	2	−64.01	YNSDLNTIR	90.91
					430.67	2	−132.91	ETDLLNR	
	CID	VM1A2_CROAD	Snake venom metalloproteinase adamalysin-2 OS = *Crotalus adamanteus* PE = 1 SV = 2	23.10	554.28	2	−23.97	YNSDLNIIR	62.52

Russell’s viper + tryptase	CID	OXLA_DABRR	l-amino-acid oxidase OS = *Daboia russelii* PE = 1 SV = 1	56.90	584.78	2	−58.23	VTVTYQTTQK	96.50
					520.58	3	−56.45	SGLTAARDVNRASEL	
	CID	IVBI2_DABRR	Protease inhibitor 2 OS = *Daboia russelii* PE = 2 SV = 1	9.70	555.73	2	−25.48	ENTNNFDTR	63.36
					677.25	2	−81.37	ENTNNFDTRDK	

Saw-scaled viper + tryptase	CID	OXLA_VIPAA	l-amino-acid oxidase OS = *Vipera ammodytes ammodytes* PE = 1 SV = 1	54.70	502.22	2	−108.63	VTVLEASER	48.50

*Samples were prepared at a ratio of 10^3^:1 venom to tryptase by mass (southern copperhead and common lancehead) or 10:1 venom to tryptase by mass (western diamondback rattlesnake, Russell’s viper, saw-scaled viper, and red spitting cobra) in PBS, incubated at 37°C for 30 min, and subsequently stored at −20°C. Controls were prepared with venom and PBS alone. Samples were analyzed by reversed phase LC-ESI-MS/MS to detect peptide biproducts of tryptase digestion. Peptides were detected for all venoms except of red spitting cobra. After incubation with tryptase, l-amino-acid oxidase peptides were detected in five different venoms. In addition, protease inhibitor and metalloproteinase peptides were detected in the tryptase-treated venom of Russell’s viper and western diamondback rattlesnake, respectively. Protein reference database was SwissProt*.

### Human Mast Cell Mediators Reduce the Mortality of Zebrafish Exposed to Lethal Doses of Cobra Venom

**Figure 6:**
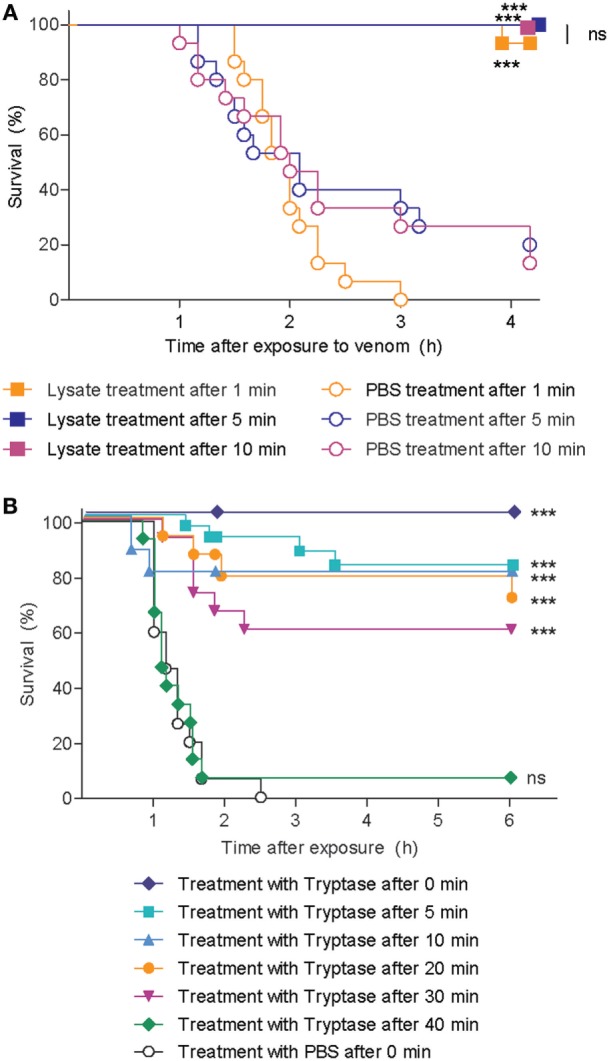
Skin mast cell lysate and purified human tryptase protects zebrafish from cobra venom when administered as a therapy. **(A)** 48 h post fertilization (hpf) zebrafish received 625 ng red spitting cobra whole venom in 100 µl each. 1, 5, or 10 min after addition of venom, zebrafish were treated with lysate from 12 thousand purified human skin mast cells per fish. Data are pooled from three independent experiments, with *n* = 4 zebrafish per group, each. **(B)** 48 hpf zebrafish received 625 ng red spitting cobra whole venom in 100 µl each. Between 0 and 40 min after addition of venom, zebrafish were treated with 10 µg/ml purified human tryptase. Data are pooled from six independent experiments with *n* = 5 embryos per group, each. Lysate, tryptase, or vehicle alone had no effect (data not shown). Kaplan–Meyer survival analysis, Log-rank (Mantel-Cox) test compares treatments to vehicle. ***p < 0.001.

To test whether human tryptase can protect against the lethal effects of snake venom when administered subsequent to initial exposure to venom, we first exposed zebrafish embryos to lethal doses of cobra venom, and then administered pooled lysate from purified hsMCs 1, 5, or 10 min later. Survival rates were significantly higher in all lysate-treated groups compared to those receiving vehicle only, with strong or complete protection of the zebrafish embryos (Figure 6A). We next performed similar experiments applying purified human tryptase 1, 5, 10, 20, 30, and 40 min following cobra venom exposure, with significantly higher survival rates for embryos that received tryptase treatment after up to 30 min when compared with embryos receiving no treatment (Figure 6B).

### Recombinant Human Tryptase β Detoxifies Snake Venom

**Figure 7:**
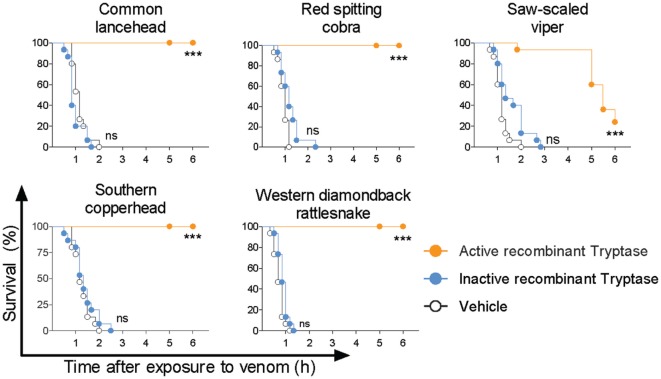
Active recombinant human tryptase β detoxifies snake venoms. Venoms were incubated with 10 µg/ml active human recombinant tryptase β and then administered to 48 h post fertilization zebrafish in 100 µl of water. Controls were inactive recombinant tryptase β and PBS (vehicle). Vehicle or enzymes alone had no effect (data not shown). Data are pooled from three to four independent experiments for *n* = 15–20 fish per group. Kaplan–Meyer survival analysis, Log-rank (Mantel-Cox) test compares treatments to vehicle. ***p < 0.001.

Purified human tryptase is only 95% pure and contains buffer components, heparin, and sodium azide as biologically active compounds. Therefore, and because recombinant human tryptase β is the likely option for the clinical treatment of snakebite, we verified the role of tryptase and the efficacy of a recombinant protease. Recombinant human tryptase β was incubated with venom from five different snakes and administered to 48 hpf zebrafish as before. Tryptase-treated lancehead, cobra, copperhead, and rattlesnake venom produced 100% survival and tryptase-treated viper venom was significantly less deadly than venom alone (Figure 7). To rule out non-enzymatic mechanisms of venom detoxification, for example, by binding of venom components to the recombinant tryptase β, we performed experiments comparing active and inactivated recombinant tryptase. Inactive recombinant human tryptase β showed no protective effect, verifying the need for an enzymatically active protease (Figure 7).

## Discussion

Currently, the only validated treatment for severe snakebite envenomation is passive immunization by i.v. infusion of animal-derived antisera, a treatment which has proven to be effective, but which is often not available for those needing it the most (31). The reasons for this include the cost of production, the need for accurate species identification, and the availability of the respective antisera. Novel treatment options that avoid these problems could be a great help in the management of snakebite envenomation. Here, we show that human tryptase β degrades and detoxifies venoms from a broad spectrum of phylogenetically distinct snakes, making recombinant tryptase a potential treatment for snakebite envenoming.

Snake fangs usually deliver venom to the dermis or in subcutaneous fat tissue, where MCs are abundant (32). Since mouse MCs are capable of reducing the toxicity of certain animal venoms and venom components (12–14), we hypothesized that human MCs can also limit venom toxicity. We show that primary hsMCs are activated to release preformed mediators upon exposure to venom from six different snake species, suggesting that human MC products may provide some evolutionarily derived defense against animal venoms, and pointing to a new pharmaceutical avenue for broadly treating snake envenoming.

To measure detoxification of snake venom by human MCs, we sought a biological toxicity assay that would allow for efficient screening of multiple treatments and venoms at various doses. Using zebrafish embryos 48 hpf, we verified that lysates from freshly isolated hsMC are sufficient to detoxify cobra venom. We proceeded to assess detoxifying effects of three purified human mast cell proteases on venom from six different snake species. We found that human tryptase is responsible for this detoxification. This is in contrast to findings in mouse models where CPA (12) and chymase (14) were found to be protective. The extent of protection by tryptase varied between venoms, likely reflecting the unique molecular composition of venom produced by different species.

Tryptase β is the predominant tryptase and serine proteinase expressed and stored in human MCs. It is active as a heparin-stabilized tetramer with trypsin-like activity sterically restricted to substrates that penetrate its central cavity to access the inward-facing active sites (33). We used MS to determine whether degradation of venom components was the mechanism of detoxification by tryptase. In order to compare protein level differences in MALDI-TOF-MS signal intensity in treated versus untreated venom, a defined quantity of horse heart myoglobin was combined with the treated venoms immediately prior to measurement to serve as an internal intensity reference. Degradation of the majority of venom protein peaks by tryptase is clearly shown by the lower relative signal intensities of the venom components exposed to tryptase (Figure 2). In addition, LC-ESI-MS/MS allowed us to identify specific peptide breakdown products from several known venom proteins found only in the tryptase-treated samples. Notably, the cleavage sites C-terminal to arginine and lysine residues in these products match the tryptic activity of tryptase β. Among the toxic venom proteins apparently degraded by tryptase were an l-amino acid oxidase and a zinc metalloproteinase. l-amino acid oxidase is an enzyme found in many snake venoms that has been shown to act on platelets and induce apoptosis, hemorrhagic effects, and cytotoxicity (30). Zinc metalloproteinases, common in crotaline and viperine snake venoms, induce capillary hemorrhage and myonecrosis, often resulting in permanent tissue loss (34). Interference with these enzymes would be an important function of therapeutic tryptase.

With only one protease responsible for detoxification of all venoms tested, the possibility of developing a simple therapy to treat snakebites from a variety of venomous snakes seems feasible. As neither MC lysates nor purified human tryptase can be used as a therapy in humans, we generated and tested recombinant human tryptase β, which could be produced in large quantities of pharmaceutical grade product. When treated with this active recombinant protease, all tested venoms were significantly reduced in toxicity, and four out of the five venoms were rendered 100% non-lethal in our toxicity assay.

Our data indicate that recombinant human tryptase β could potentially be used as a first aid treatment for snakebite envenoming. A major advantage of such an approach is that treatment could be initiated immediately after a bite without identification of the snake species or access to the species-specific antidote, and would not require intravenous administration. Applied locally within a short time it could reduce lasting tissue damage and minimize the spread of venom while the victim is transported to a medical facility for further treatment. A unique feature of tryptase makes this treatment approach likely to be very well tolerated: in contrast to any other serine protease, tryptase is active as a tetramer and the access to the four active sites of the tetramer is very limited as they are directed toward its central pore (35). This special feature of tryptase prevents it, for example, from degrading larger proteins in the skin which would otherwise lead to severe damage of the connective tissue.

Several aspects of therapeutic use of tryptase or protease with tryptase-like activity require further investigation. (1) Both the overall detoxifying ability of tryptase and the therapeutic approach have so far only been tested in the zebrafish embryo model. Even though our proteomics approach allowed us to verify the degradation of certain venom toxins, different toxins, perhaps with increased resistance to tryptase digestion could be responsible for toxicity in humans. (2) Although tryptase is locally released in the skin by degranulation of MCs without any pathological consequences, and despite the above-described unique property of tryptase acting as a tetramer, larger amounts of locally administered tryptase β could theoretically lead to adverse events. (3) Finally, because active tryptase in working concentrations is rapidly degraded and loses its activity, methods and mechanisms of storing and applying the enzyme in the field need to be developed.

## Author Contributions

All authors have contributed to the collection, analysis, or interpretation of data and have contributed to drafting of the manuscript. EA and MaMe have written the manuscript, figures were made by EA and KS, study conception by MaMe, study design by EA, MaMa, and MaMe.

## Conflict of Interest Statement

The authors declare that the research was conducted in the absence of any commercial or financial relationships that could be construed as a potential conflict of interest. The reviewer NG and handling Editor declared their shared affiliation.
