# Supplementary material for: Human Mast Cell Tryptase Is a Potential Treatment for Snakebite Envenoming Across Multiple Snake Species
Source: Front Immunol. 2018 Jul 9;9:1532. doi: 10.3389/fimmu.2018.01532 (PMC6047305; doi:10.3389/fimmu.2018.01532)
Supplement: Supplementary file 1 [file Data_Sheet_1.pdf]

Project Info

Name: Venom protein identification 26082013 **Date:** August 26, 2013

Sample Info & Protocols

Date: August 26, 2013

Name: **Southern Copperhead + Tryptase**

Search Result Info

Search Result	Location	Search Engine	Database	Ident. Compounds
Amazon vertebrates_swissprot july_Mascot_2013-08-28 18:47:18	/Venom protein identification 26082013/Acc + T 3rd batch 5 ul_RC5_01_2660.d/ProteinAnalysisResults.mgf	Mascot, 2.3.02	SwissProt_July2013, uniprotsprotJuly2013.fasta	3/501 (FDR:0.00%)

Protein 1: L-amino-acid oxidase (Fragment) OS=Bothrops jararacussu PE=1 SV=1

Accession: OXLA_BOTJR **Score:** 70.21

Database: SwissProt_July2013 **MW [kDa]:** 56.30

Seq. Coverage [%]: 2.40 % **pI:** 5.73

No. of Peptides: 2

10	20	30	40	50	60	70	80	90	100	110	120
MNVFFMFSKP	GKLADDRNPL	EECFRETDYE	EFLEIAKNGL	STTSNPKRVV	IVGAGMSGLS	AAYVLANAGH	QVTVLEASER	AGGQVKTYRN	EKEGWYANLG	PMRLPEKHRI	VREYIRKFGL
130	140	150	160	170	180	190	200	210	220	230	240
QLNEFSQENE	NAWYFIKNIR	KRVGEVNKDP	GVLDYPVKPS	EVGKSAGQLY	EESLQKAVEE	LRRTNCSYML	NKYDTYSTKE	YLLKEGNLSP	GAVDMIGDLL	NEDSGYYVSF	IESLKHDDIF
250	260	270	280	290	300	310	320	330	340	350	360
AYEKRFDEIV	GGMDKLPTSM	YQAIQEKVHL	NARVIKIQQD	VKEVTVTYQT	SEKETLSVTA	DYVIVCTTSR	AARRIKFEPP	LPPKKAHALR	SVHYRSGTKI	FLTCTKKFWE	DDGIHGGKST
370	380	390	400	410	420	430	440	450	460	470	480
TDLPSRFIYY	PNHNFPNGVG	VIIAYGIGDD	ANYFEALDFE	DCGDIVINDL	SLIHQLPKEE	IQAICRPSMI	QRWSLDKYAM	GGITTFTPYQ	FQHFSEALTA	PVDRIYFAGE	YTAQAHGWIA
490	500										
STIKSGPEGL	DVNRASE										

Cmpd.	No. of Cmpds.	m/z meas.	Δ m/z [ppm]	z	Rt [min]	Score	P	Range	Sequence	Modification
148	1	441.3400	155.90	3	29.0	27.7	2	314-324	R.RIKFEPPLPPK.K	
137	2	432.0000	148.58	3	27.8	26.9	2	315-325	R.IKFEPPLPPKK.A	

BRUKER

Project Info

**Name:**	Venom protein identification 26082013	**Date:**	August 26, 2013

Sample Info & Protocols

**Name:**	**Common lancehead + Tryptase**	**Date:**	August 26, 2013

Search Result Info

Search Result	Location	Search Engine	Database	Ident. Compounds
Amazon vertebrates_swissprot july_Mascot_2013-08-28 18:47:18	/Venom protein identification 26082013/Ba + T 2nd batch 7 ul_BA5_01_2683.d/ProteinAnalysisResults.mgf	Mascot, 2.3.02	SwissProt_July2013, uniprotsprotJuly2013.fasta	4/501 (FDR:0.00%)

Protein 1: L-amino-acid oxidase (Fragment) OS=Bothrops jararacussu PE=1 SV=1

**Accession:**	OXLA_BOTJR	**Score:**	146.69
**Database:**	SwissProt_July2013	**MW [kDa]:**	56.30
**Seq. Coverage [%]:**	6.20 %	**pI:**	5.73
		**No. of Peptides:**	4

10	20	30	40	50	60	70	80	90	100	110	120
MNVFFMFSKP	GKLADDRNPL	EECFRETDYE	EFLEIAKNGL	STTSNPKRVV	IVGAGMSGLS	AAYVLANAGH	QVTVLEASER	AGGQVKTYRN	EKEGWYANLG	PMRLPEKHRI	VREYIRKFGL
130	140	150	160	170	180	190	200	210	220	230	240
QLNEFSQENE	NAWYFIKNIR	KRVGEVNKDP	GVLDYPVKPS	EVGKSAGQLY	EESLQKAVEE	LRRTNCSYML	NKYDTYSTKE	YLLKEGNLSP	GAVDMIGDLL	NEDSGYYVSF	IESLKHDDIF
250	260	270	280	290	300	310	320	330	340	350	360
AYEKRFDEIV	GGMDKLPTSM	YQAIQEKVHL	NARVIKIQQD	VKEVTVTYQT	SEKETLSVTA	DYVIVCTTSR	AARRIKFEPP	LPPKKAHALR	SVHYRSGTKI	FLTCTKKFWE	DDGIHGGKST
370	380	390	400	410	420	430	440	450	460	470	480
TDLPSRFIYY	PNHNFPNGVG	VIIAYGIGDD	ANYFEALDFE	DCGDIVINDL	SLIHQLPKEE	IQAICRPSMI	QRWSLDKYAM	GGITTFTPYQ	FQHFSEALTA	PVDRIYFAGE	YTAQAHGWIA
490	500										
STIKSGPEGL	DVNRASE										

Cmpd.	No. of Cmpds.	m/z meas.	Δ m/z [ppm]	z	Rt [min]	Score	P	Range	Sequence	Modification
165	2	441.3500	178.56	3	28.2	34.7	2	314-324	R.RIKFEPPLPPK.K	
114	1	463.6000	90.68	3	22.5	47.9	1	347-358	K.KFWEDDGIHGGK.S	
125	1	420.9300	167.17	3	23.6	37.1	0	348-358	K.FWEDDGIHGGK.S	
57	1	438.7800	126.03	2	15.9	27.0	0	359-366	K.STTDLPSR.F	

Project Info

Name: venom protein identification 27082013 **Date:** August 27, 2013

Sample Info & Protocols

Name: Western diamondback rattlesnake + Tryptase **Date:** August 27, 2013

Search Result Info

Search Result	Location	Search Engine	Database	Ident. Compounds
Amazon vertebrates_swissprot july_Mascot_2013-08-28 18:47:40	/venom protein identification 27082013 /Ca + Tryptase_II_BE2_01_2132.d/Ca + Tryptase_II_BE2_01_2132.mgf	Mascot, 2.3.02	SwissProt_July2013, uniprotsprotJuly2013.fasta	6/484 (FDR:0.00%)

Protein 1: Snake venom metalloproteinase atrolysin-C OS=Crotalus atrox PE=1 SV=1

Accession: VM1AC_CROAT **Score:** 90.91

Database: SwissProt_July2013 **MW [kDa]:** 46.70

Seq. Coverage [%]: 3.90 % **pI:** 4.85

No. of Peptides: 2

```
        10         20         30         40         50         60         70         80         90        100        110        120
MIEVVLVTIC LAVFPYQGSS IILESGNVND YEVVYPRKVT ALPKGAVQPK YEDAMQYELK VNGEPVVLHL EKNKELFSKD YSETHYSPDG RKITTNPSVE DHCYYRGRIE NDADSTASIS
       130        140        150        160        170        180        190        200        210        220        230        240
ACNGLKGHFK LQGELYLIEP LELSDSEAHA VFKLENVEKE DEAPKMCGVT QNWESYEPIK KASDLNLNPD QQNLPQRYIE LVVVADHRVF MKYNSDLNTI RTRVHEIVNF INGFYRSLNI
       250        260        270        280        290        300        310        320        330        340        350        360
HVSLTDLEIW SNEDQINIQS ASSDTLNAFA EWRETDLLNR KSHDNAQLLT AIELDEETLG LAPLGTMCDP KLSIGIVQDH SPINLLMGVT MAHELGHNLG MEHDGKDCLR GASLCIMRPG
       370        380        390        400        410        420
LTKGRSYEFS ADSMHYYERF LKQYKPQCIL NKPLRIDPVS TPVSGNELLE AGEE
```

Cmpd.	No. of Cmpds.	m/z meas.	Δ m/z [ppm]	z	Rt [min]	Score	P	Range	Sequence	Modification
98	1	548.2400	-64.01	2	22.2	64.5	0	213-221	K.YNSDLNTIR.T	
80	1	430.6700	-132.91	2	21.3	26.4	0	274-280	R.ETDLLNR.K	

L-amino-acid oxidase OS=Crotalus adamanteus PE=1 SV=1

Protein 2:

**Accession:**	OXLA_CROAD	**Score:**	65.46
**Database:**	SwissProt_July2013	**MW [kDa]:**	58.60
**Seq. Coverage [%]:**	3.30 %	**pI:**	6.32
		**No. of Peptides:**	2

10	20	30	40	50	60	70	80	90	100	110	120
MNVFFMFSLL	FLAALGSCAH	DRNPLEECFR	ETDYEEFLEI	AKNGLTATSN	PKRVVIVGAG	MAGLSAAYVL	AGAGHQVTVL	EASERVGGRV	RTYRKKDWYA	NLGPMRLPTK	HRIVREYIKK
**130**	**140**	**150**	**160**	**170**	**180**	**190**	**200**	**210**	**220**	**230**	**240**
FDLKLNEFSQ	ENENAWYFIK	NIRKRVREVK	NNPGLLEYPV	KPSEEGKSAA	QLYVESLRKV	VEELRSTNCK	YILDKYDTYS	TKEYLLKEGN	LSPGAVDMIG	DLLNEDSGYY	VSFIESLKHD
**250**	**260**	**270**	**280**	**290**	**300**	**310**	**320**	**330**	**340**	**350**	**360**
DIFGYEKRFD	EIVGGMDQLP	TSMYEAIKEK	VQVHFNARVI	EIQQNDREAT	VTYQTSANEM	SSVTADYVIV	CTTSRAARRI	KFEPPLPPKK	AHALRSVHYR	SGTKIFLTCT	KKFWEDDGIH
**370**	**380**	**390**	**400**	**410**	**420**	**430**	**440**	**450**	**460**	**470**	**480**
GGKSTTDLPS	RFIYYPNHNF	TSGVGVIIAY	GIGDDANFFQ	ALDFKDCADI	VINDLSLIHE	LPKEDIQTFC	HPSMIQRWSL	DKYAMGGITT	FTPYQFQHFS	EALTAPFKRI	YFAGEYTAQF
**490**	**500**	**510**	**520**								
HGWIDSTIKS	GLTAARDVNR	ASENPSGIHL	SNDNEF								

Cmpd.	No. of Cmpds.	m/z meas.	Δ m/z [ppm]	z	Rt [min]	Score	P	Range	Sequence	Modification
71	2	557.7900	-11.11	2	20.0	35.7	0	279-287	R.VIEIQQNDR.E	
21	3	438.6800	-101.90	2	16.1	29.8	0	364-371	K.STTDLPSR.F	

Protein 3: Snake venom metalloproteinase adamalysin-2 OS=Crotalus adamanteus PE=1 SV=2

**Accession:**	VM1A2_CROAD	**Score:**	62.52
**Database:**	SwissProt_July2013	**MW [kDa]:**	23.10
**Seq. Coverage [%]:**	4.40 %	**pI:**	9.58
		**No. of Peptides:**	1

```
         10         20         30         40         50         60         70         80         90        100        110        120
QQNLPQRYIE LVVVADRRVF MKYNSDLNII RTRVHEIVNI INGFYRSLNI DVSLVNLEIW SGQDPLTIQS SSSNTLNSEG LWREKVLLNK KKKDNAQLLT AIEFKCETLG KAYLNSMCNP
        130        140        150        160        170        180        190        200        210
RSSVGIVKDH SPINLLVAVT MAHELGHNLG MEHDGKDCLR GASLCIMRPG LTPGRSYEFS DDSMGYYQKF LNQYKPQCIL NKP
```

Cmpd.	No. of Cmpds.	m/z meas.	Δ m/z [ppm]	z	Rt [min]	Score	P	Range	Sequence	Modification
204	2	554.2800	-23.97	2	29.7	62.5	0	23-31	K.YNSDLNIIR.T	

Project Info

Name: venom protein identification 27082013 **Date:** August 27, 2013

Sample Info & Protocols

Name: **Russell's viper + Tryptase** **Date:** August 27, 2013

Search Result Info

Search Result	Location	Search Engine	Database	Ident. Compounds
Amazon vertebrates_swissprot july_Mascot_2013-08-28 18:47:40	/venom protein identification 27082013 /Dr + Tryptase_II_BE4_01_2134.d/Dr + Tryptase_II_BE4_01_2134.mgf	Mascot, 2.3.02	SwissProt_July2013, uniprotsprotJuly2013.fasta	4/501 (FDR:0.00%)

Protein 1: L-amino-acid oxidase OS=Daboia russelii PE=1 SV=1

Accession: OXLA_DABRR **Score:** 96.50

Database: SwissProt_July2013 **MW [kDa]:** 56.90

Seq. Coverage [%]: 5.00 % **pI:** 8.55

No. of Peptides: 2

10	20	30	40	50	60	70	80	90	100	110	120
MNVFFMFSLL	FLATLGSCAD	DKNPLEECFR	EDDYEEFLEI	AKNGLKKTSN	PKHIVIVGAG	MSGLSAAYVL	AGAGHKVTVL	EASERPGGRV	RTHRNVKEGW	YANLGPMRVP	EKHRIIREYI
130	140	150	160	170	180	190	200	210	220	230	240
RKFGLKLNEF	VQETENGWYF	IKNIRKRVGE	VKKDPGLLKY	PVKPSEAGKS	AGQLYQESLG	KAVEELKRTN	CSYILNKYDT	YSTKEYLIKE	GNLSPGAVDM	IGDLLNEDSG	YYVSFIESLK
250	260	270	280	290	300	310	320	330	340	350	360
HDDIFAYEKR	FDEIVGGMDQ	LPTSMYRAIE	ESVHFKARVI	KIQQNAEKVT	VTYQTTQKNL	LLETADYVIV	CTTSRAARRI	TFKPPLPPKK	AHALRSVHYR	SGTKIFLTCT	KKFWEDDGIQ
370	380	390	400	410	420	430	440	450	460	470	480
GGKSTTDLPS	RFIYYPNHNF	TTGVGVIIAY	GIGDDANFFQ	ALNLNECADI	VFNDLSSIHQ	LPKKDLQTFC	YPSIIQKWSL	DKYAMGAITT	FTPYQFQHFS	EALTAPVGRI	FFAGEYTANA
490	500	510									
HGWIDSTIKS	GLTAARDVNR	ASEL									

Cmpd.	No. of Cmpds.	m/z meas.	Δ m/z [ppm]	z	Rt [min]	Score	P	Range	Sequence	Modification
50	2	584.7800	-58.23	2	18.7	49.9	0	289-298	K.VTVTYQTTQK.N	
147	1	520.5800	-56.45	3	24.9	30.8	2	490-504	K.SGLTAARDVNRASEL.-	

**Protein 2:**	Protease inhibitor 2 OS=Daboia russelii PE=2 SV=1		
**Accession:**	IVBI2_DABRR	**Score:**	63.36
**Database:**	SwissProt_July2013	**MW [kDa]:**	9.70
**Seq. Coverage [%]:**	13.10 %	**pI:**	9.84
		**No. of Peptides:**	2

```
        10         20         30         40         50         60         70         80         90
MSSGGLLLLL GLLTLWAEPT PISGQDRPKF CFLRPDFGRY GHPRPRFYYN PATNQCQGFL AQRSRENTNN FDTRDKCRQT CGRK
```

Cmpd.	No. of Cmpds.	m/z meas.	Δ m/z [ppm]	z	Rt [min]	Score	P	Range	Sequence	Modification
19	4	555.7300	-25.48	2	15.0	41.5	0	66-74	R.ENTNNFDTR.D	
12	1	677.2500	-81.37	2	13.5	23.1	1	66-76	R.ENTNNFDTRDK.C	

Project Info

Name: venom protein identification 27082013 **Date:** August 27, 2013

Sample Info & Protocols

Date: August 27, 2013

Name: Saw-scaled viper + Tryptase

Search Result Info

Search Result	Location	Search Engine	Database	Ident. Compounds
Amazon vertebrates_swissprot july_Mascot_2013-08-28 18:47:40	/venom protein identification /Ec + Tryptase_II_BE6_01_2137.d/Ec + Tryptase_II_BE6_01_2137.mgf	Mascot, 2.3.02	SwissProt_July2013, uniprotsprotJuly2013.fasta	1/445 (FDR:0.00%)

Protein 1: L-amino-acid oxidase OS=Vipera ammodytes ammodytes PE=1 SV=1

Accession: OXLA_VIPAA **Score:** 48.50

Database: SwissProt_July2013 **MW [kDa]:** 54.70

Seq. Coverage [%]: 1.90 % **pI:** 7.71

No. of Peptides: 1

```
        10         20         30         40         50         60         70         80         90        100        110        120
DRNPLEECFR ETDYEEFLEI ARNGLKKTSN PKHVVVVGAG MSGLSAAYVL AGAGHKVTVL EASERAGGRV RTHRNSKEGW YANLGPMRIP EKHRIVREYI RKFGLNLNEF SQENDNAWYF
       130        140        150        160        170        180        190        200        210        220        230        240
IKNIRKRVGE VNKDPGLLKY PVKPSEEGKS AGQLYEESLG SAVKDLKRTN CSYILNKYDT YSTKEYLIKE GNLSPGAVDM IGDLLNEDSG YYVSFIESLK HDDIFAYEKR FDEIVGGMDQ
       250        260        270        280        290        300        310        320        330        340        350        360
LPTSMYRAIE EKVKFNARVI KIQQNANQVT VTYQTPEKDT SSNTADYVIV CTTSRAARRI QFEPPLPPKK QHALRSVHYR SGTKIFLTCS SKFWEDDGIH GGKSTTDLPS RFIYYPNHNF
       370        380        390        400        410        420        430        440        450        460        470        480
STGVGVIIAY GIGDDANFFQ ALKFKDCADI VFNDLSLIHQ LPKEEIQSFC YPSMIQKWSL DKYAMGAITT FTPYQFQRFS EALTAPQGRI FFAGEYTAEA HGWIDSTIKS GLTAARDVNR
       490
ASEQ
```

Cmpd.	No. of Cmpds.	m/z meas.	Δ m/z [ppm]	z	Rt [min]	Score	P	Range	Sequence	Modification
62	3	502.2200	-108.63	2	22.1	48.5	0	57-65	K.VTVLEASER.A	
